# Piped water supply interruptions and acute diarrhea among under-five children in Addis Ababa slums, Ethiopia: A matched case-control study

**DOI:** 10.1371/journal.pone.0181516

**Published:** 2017-07-19

**Authors:** Metadel Adane, Bezatu Mengistie, Girmay Medhin, Helmut Kloos, Worku Mulat

**Affiliations:** 1 Ethiopian Institute of Water Resources (EIWR), Addis Ababa University, Addis Ababa, Ethiopia; 2 College of Health and Medical Sciences, Haramaya University, Haramaya, Ethiopia; 3 Aklilu Lemma Institute of Pathobiology, Addis Ababa University, Addis Ababa, Ethiopia; 4 Department of Epidemiology and Biostatistics, University of California, San Francisco, California, United States of America; 5 Department of Civil and Environmental Engineering, University of Connecticut, Storrs, CT, United States of America; University of Otago, NEW ZEALAND

## Abstract

**Background:**

The problem of intermittent piped water supplies that exists in low- and middle-income countries is particularly severe in the slums of sub-Saharan Africa. However, little is known about whether there is deterioration of the microbiological quality of the intermittent piped water supply at a household level and whether it is a factor in reducing or increasing the occurrence of acute diarrhea among under-five children in slums of Addis Ababa. This study aimed to determine the association of intermittent piped water supplies and point-of-use (POU) contamination of household stored water by *Escherichia coli (E*. *coli)* with acute diarrhea among under-five children in slums of Addis Ababa.

**Methods:**

A community-based matched case-control study was conducted from November to December, 2014. Cases were defined as under-five children with acute diarrhea during the two weeks before the survey. Controls were matched by age and neighborhood with cases by individual matching. Data were collected using a pre-tested structured questionnaire and *E*. *coli* analysis of water from piped water supplies and household stored water. A five-tube method of Most Probable Number (MPN)/100 ml standard procedure was used for *E*. *coli* analysis. Multivariable conditional logistic regression with 95% confidence interval (CI) was used for data analysis by controlling potential confounding effects of selected socio-demographic characteristics.

**Main findings:**

During the two weeks before the survey, 87.9% of case households and 51.0% of control households had an intermittent piped water supply for an average of 4.3 days and 3.9 days, respectively. POU contamination of household stored water by *E*. *coli* was found in 83.3% of the case households, and 52.1% of the control households. In a fully adjusted model, a periodically intermittent piped water supply (adjusted matched odds ratio (adjusted mOR) = 4.8; 95% CI: 1.3–17.8), POU water contamination in household stored water by *E*. *coli* (adjusted mOR = 3.3; 95% CI: 1.1–10.1), water retrieved from water storage containers using handle-less vessels (adjusted mOR = 16.3; 95% CI: 4.4–60.1), and water retrieved by interchangeably using vessels both with and without handle (adjusted mOR = 5.4; 95% CI: 1.1–29.1) were independently associated with acute diarrhea.

**Conclusion:**

We conclude that provision of continuously available piped water supplies and education of caregivers about proper water retrieval methods of household stored water can effectively reduce POU contamination of water at the household level and thereby reduce acute diarrhea among under-five children in slums of Addis Ababa. Promotion of household water treatment is also highly encouraged until the City’s water authority is able to deliver continuously available piped water supplies.

## Introduction

One hundred forty-seven countries, including Ethiopia, met the 2015 Millennium Development target of providing drinking water from improved sources [1]. However, a recent study indicated that policies based on monitoring of progress toward Millennium Development Goals (MDGs) and ongoing Sustainable Development Goals (SDGs) failed to consider the range of challenges that have yet to be solved in order to meet people’s water and sanitation needs, and therefore give an inflated sense of progress [2]. Although several studies have documented that water from improved sources reduced the occurrence of diarrhea among under-five children [3–5], less information is known about the safety [6] and microbial quality of water [6, 7]. This discrepancy is in large extent due to the intermittent nature of piped water supplies from improved sources in many low- and middle-income countries, where over one-third of urban water supplies are frequently interrupted [8].

A recent review estimated that at least 309 million people worldwide experience interruption of their water supplies [9]. Intermittent water supplies transmit waterborne pathogens [10], increase household water storage times [11–13], and jeopardize hygiene practices [12, 13]. Furthermore, wide-mouthed water storage containers are vulnerable to contamination by unclean hands, cups, and other water retrieval vessels [14–16]. A recent systematic review revealed one study that reported a 73.0% reduction in diarrhea after the change from intermittent to continuous water supplies [17]. Another systematic review by Fewtrell et al. [18] revealed that improvements of the microbiological quality of water reduced the risk of diarrhea-related morbidity by 31.0%. Safe storage of water at the household level [3, 19–21] and continuous availability of improved water supply [10, 22] have been identified as effective measures to prevent diarrhea. Safe water storage, proper water-handling practices and household level water treatment have been reported to reduce the risk of diarrhea by 25.0% - 85.0% [20, 21]. Hence, safe piped water supplies alone may not be a guarantee for preventing diarrhea because of potential issues arising from a lack of continuous availability and microbiological contamination of water through poor household water-handling practices [3, 23].

Although *E*. *coli* is an indicator of fecal contamination of water as a cause or control of diarrhea [24, 25], the epidemiology of intermittent water supplies needs to be better understood [9, 26]. Urban areas in Ethiopia are growing rapidly, with slums covering about 80.0% of Addis Ababa in 2010 [27]. Intermittent piped water supplies present an ongoing problem in Addis Ababa, especially in the slums, despite significant efforts made by the City’s Water Authority. Thus, it is unknown if the availability of piped water in Addis Ababa slums ensures the consumption of clean water and reduces acute diarrhea among under-five children.

This study examines intermittent piped water supplies, household water-handling practices, and POU water contamination by *E*. *coli* in household stored water in relation to acute diarrhea among under-five children in slums of Addis Ababa. The results of this study may assist Ethiopian water supply officials and health policy makers in designing programs for continuous availability of water to reduce the incidence of acute diarrhea among under-five children in slums of Addis Ababa and other urban slums in Ethiopia.

## Materials and methods

### Study area

The study was conducted in seven slum *kebeles* of Addis Ababa (three *kebeles* in Gullele Sub-City in District (W*oreda*) 01 and four *kebeles* in Lideta Sub-City in District 05). *Kebeles* were the lowest administrative structures in Addis Ababa before 2011; since then, the Districts have become the lowest administrative structures. This study used both *kebele and* District designations to be consistent with the existing literature. The Addis Ababa Water and Sewerage Authority is responsible for providing piped water supplies from improved sources, that is piped water mainly from surface water in the nearby Legadadi, Dire, and Gefersa reservoirs, and also groundwater pumped from the Akaki Wellfield [28].

Interruption of the water supply occurs in Addis Ababa mainly because of low capacity of water production (due to scarcity of surface water for the water treatment plants during the dry and short rainy seasons), the old and poorly maintained distribution system, mechanical failure and thus need of frequent interruption for repair of distribution systems [28]. Since occurrences of intermittent stoppage of the water supplies are often unknown ahead of time (unless the City’s water supply authority has a planned outage and makes an announcement ahead of time through the public media), householders are inclined to store water in anticipation of future stoppages.

### Study design and period

A community-based matched case-control study design was employed from November to December, 2014. Cases were defined as under-five children with acute diarrhea during the two weeks before the survey, whereas controls were under-five children without acute diarrhea during the two weeks before the survey. Controls were matched to cases by age and neighborhood using individual matching.

### Sample size calculation

Sample size was calculated using matched case-control study design sample size estimation methods [29] by considering Pitman efficiency assumption of matched pair sample size [30]. A matched sample size of 199 pairs using individual matching (1 case to 3 controls; which means 199 cases:597 controls) was considered adequate by assuming 1) a probability of type I error 5%; 2) a 90% power of the test; 3) a probability of type II error 10%; 4) a proportion (p) of POU contamination of household stored water by *E*. *coli* among controls compared to piped water supply is 41.8% [31]; 5) expected odds ratio is 1.75; 6) a 10% non-response rate; and 7) control-to-case ratio (r) is 3, based on Pitman efficiency criteria [32]. Twenty-five percent matched pair samples of case and control households (50 cases:150 controls) were taken as adequate for water samples in household stored water and from piped water supply. This 25% out of the total matched pair for water samples was determined based on similar studies [31, 33].

### Inclusion and exclusion criteria

Children under five years of age with acute diarrhea during the two weeks before the preliminary survey might become free of acute diarrhea during the survey (and also vice versa), and thus were not considered as cases. When there was more than one acute diarrheal under-five child in a household, one of the children was randomly selected before interviewing the caregiver. During selection of cases and matched controls, households having under-five children who had fetched water from piped water supplies during the two weeks before the survey were included. During water sampling from direct-piped water supplies and household stored water, households were excluded if they did not store water during the survey time. Under-five children with bloody and/or persistent diarrhea during the two weeks before the survey were excluded because of dysentery being a frequent cause of bloody diarrhea and the 14-days or longer duration of persistent diarrhea.

### Case and control selection

#### Cases

There was no specific sampling frame for selecting cases using systematic sampling techniques because daily fluctuations in specific cases of acute diarrhea would make the exact number of cases in each District or *kebele* during the two months of the survey period unknown. However, for obtaining representative cases in each slum District, we performed a preliminary survey. All houses with children aged 0–59 months with acute diarrhea (cases) were enumerated to determine the sampling population of cases in the two Districts (seven *kebeles)*. Then, sample size for cases was proportionally allocated for each slum *kebele*. Finally, during the survey, data enumerators identified cases during house-to-house transect walks in each *kebele* until the proportionally allocated sample size was achieved.

During the selection of cases, diarrhea was identified using World Health Organization (WHO) [34] signs and symptoms for diarrhea. WHO [34] defines diarrhea as the passage of three or more abnormally loose, watery or liquid stools per day. However, the WHO definition does not specify the recall period and the types of diarrhea. In our study, cases were considered to be only acute diarrhea using a two week recall period as specified in the World Gastroenterology Organization global guidelines for acute diarrhea [35].

#### Controls

During the selection of controls, control children were matched for age and neighborhood with cases. Individual matching was carried out one case at a time by selecting three controls from the immediate (closest) neighborhood first and then continued with houses having under-five children in house-to-house visits during transect walks until all controls matched all cases. During the matching of controls by neighborhood, no effort was made to identify houses immediately adjacent; however, cases and controls were in the same *kebele*. The age of the control was based on the three age categories: control age = case age ± 2 months for infants (0–11 months), ± 3 months for toddlers (12–23 months), and ± 6 months for children (24–59 months). These three age categories of control selection criteria were consistent with some studies [36, 37]. We used the three age categories of matching for availability of controls within similar or closely similar age with cases.

### Data collection

**Fig 1:**
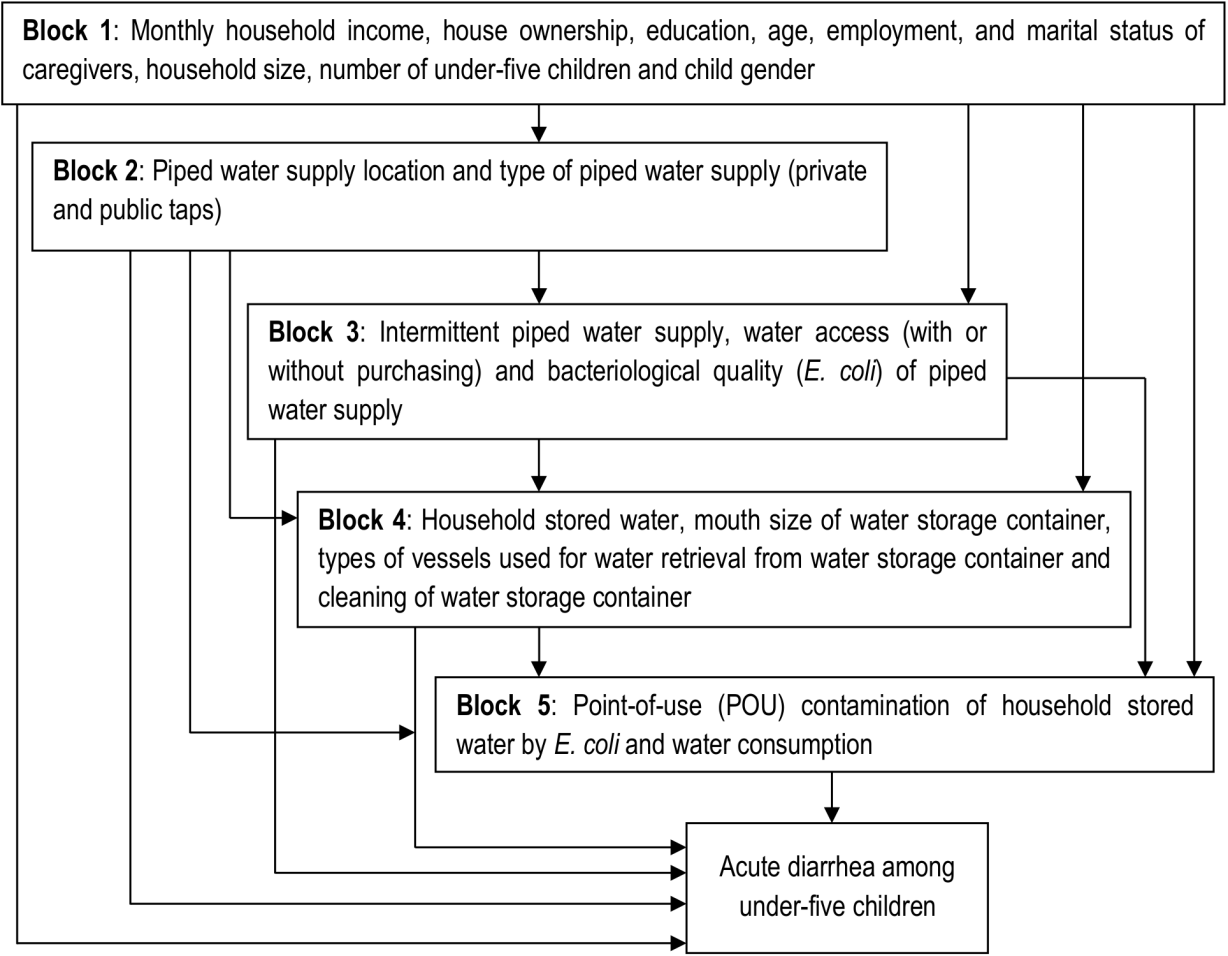
A conceptual hierarchical framework for risk factors POU contamination of household stored water by E. coli in Addis Ababa slums, Ethiopia, November to December, 2014.

Household survey data were collected from November to December, 2014 on socio-demographic and water-related factors, which were predefined in the conceptual framework (Fig 1). Some variables representing these factors were also coded using the operational definitions given in Table 1. For both case and control households, seven trained female nurses and environmental health professionals administered the survey by interviewing primary caregivers (mothers) using a pre-tested structured questionnaire. *E*. *coli* analysis of water from piped water supplies (private and public [tanker and *Bono*] taps) and household stored water was performed in the Addis Ababa Water and Sewerage Authority bacteriological laboratory using five-tube MPN/100 ml methods.

**Table 1:** Operational definitions for coding variables included in the analysis.

Variables	Operational definitions
Wide- or narrow-mouthed water storage container	Wide-mouthed (≥6 cm) and narrow-mouthed (<6 cm) [22]. Wide-mouthed indicated a water storage container that a hand could enter when dipping for water with a water retrieval vessel.
Water retrieving vessel having handle or without handle	Retrieving water from water storage container either by dipping or pouring using a vessel (cup, ladle and other) having handle or without handle.
Intermittent piped water supply	There was no continuous availability of water from piped water during the two weeks before the survey.
Piped water supply	Municipally piped water at private and public taps [40]. In this study, public water taps included *Bono* and tanker taps, whereas a private tap was owned by one household, but was also used by one or more other households.
POU contamination of household stored water *by E*. *coli*	POU contamination of household stored water was indicated by a greater number of *E*. *coli* positive tubes in household stored water compared to the respective piped water supply where the water had been fetched. However, if there were the same number of *E*. *coli* positive tubes or zero positive tubes in the household stored water and direct-piped water supply; it was taken as no POU contamination of household stored water *by E*. *coli*.
Cleaning of water storage container	Washing of water storage container once during the two weeks before the survey.
Daily per capita water consumption (l/c/d)	Quantity of water consumed per day by each household member during the two weeks before the survey.
Five tubes MPN/100 ml	0 positive tube*(<2.2 *E*. *coli* MPN/100 ml), 1 positive tube (2.2 *E*. *coli* MPN/100 ml), 2 positive tubes (5.1 *E*. *coli* MPN/100 ml), 3 positive tubes (9.2 *E*. *coli* MPN/100 ml), 4 positive tubes (16 *E*. *coli* MPN/100 ml) and 5 positive tubes (>16 *E*. *coli* MPN/100 ml) [39].

*E*. *coli*, *Escherichia coli*; l/c/d, liter per capita per day; MPN, Most Probable Number; POU, Point-of-Use

*Complies with bacteriological drinking water quality standards [no positive *E*. *coli* tube detected (<2.2 *E*. *coli* MPN/100 ml)] of WHO and Ethiopia [41, 42].

#### Water sample collection

**Fig 2:**
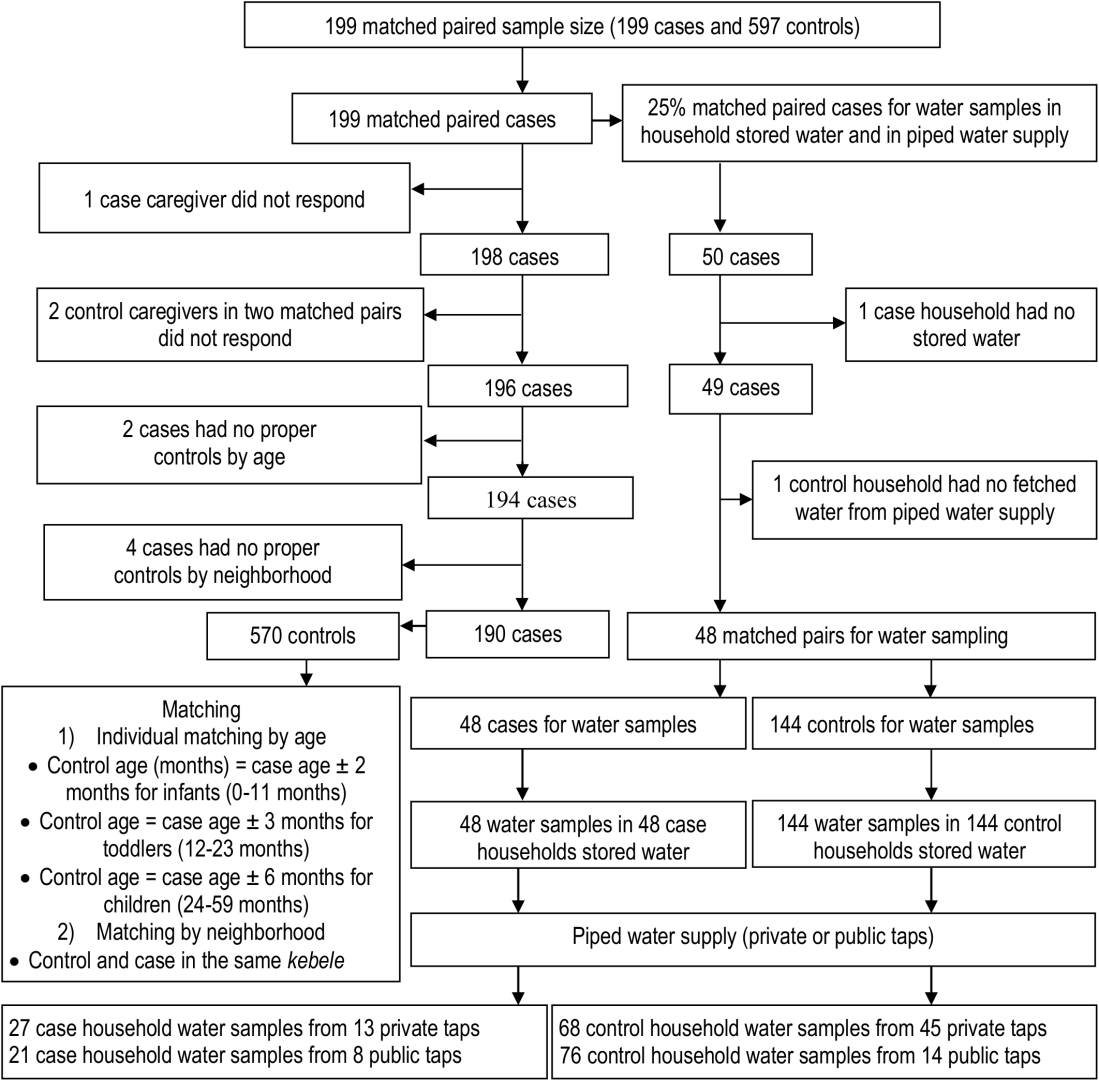
A community-based individual matched case-control study flow chart of Addis Ababa slums, Ethiopia, November to December, 2014.

Every fourth case household was selected for water sampling. Water samples were also taken from the matched control households. When one water sample was taken in a case household, three water samples were also taken from the three matched control households. In households with more than one water storage container, the container to be water sampled was randomly selected. The type of piped water taps was identified by asking each caregiver for the source from which the water had been fetched during the two weeks before the survey. If both case and control households had fetched water from the same piped water supply, only one water sample was taken (Fig 2).

All water samples were collected using sterile polyethylene water sampling bottles (250 ml). Collected water samples were stored in an ice-box and transported to the Addis Ababa Water and Sewerage Authority’s bacteriological laboratory within 2 hrs of collection for laboratory analysis within 2 to 4 hrs. Water sampling procedures and transport of water samples to laboratory rooms followed WHO guidelines [38] (S1). The number of water samples taken from piped water supply and household water storage containers is described in Fig 2. Water samples for cases and controls were taken concurrently from the household stored water and their direct-piped water supply.

#### Water analysis for *E*. *coli*

A five-tube method of MPN/100 ml standard procedure was used for *E*. *coli* analysis of piped water supplies and stored water. For lactose broth media preparations, 13 grams of lactose broth medium powder were mixed thoroughly in l liter of distilled water and dispensed in 10 ml increments into a hundred 10 ml tubes containing Durham tubes and then autoclaved at 121^°^C for 15 minutes at 15 pounds of pressure per square inch (psi). For *E*. *coli* liquid media preparations, 37 grams of *E*. *coli* broth medium powder were mixed thoroughly in l liter of distilled water and dispensed in increments of 10 ml into a hundred 10 ml tubes containing Durham tubes and then autoclaved at 121^°^C for 15 minutes [39].

For the growth of bacteria, a presumptive phase of inoculation of 10 ml of water sample for each 5 tubes containing the prepared lactose media was dispensed and incubated in an incubator at 44.5^°^C for 24 to 48 hrs. Tubes with gas bubble formation and/or cloudy color within 24 hrs were positive for bacteria and the remaining negative tubes were kept for additional 24 hrs. For confirmation of the presence of *E*. *coli* from positive tubes, *E*. *coli* selective media was used. 10 ml from 20 ml of lactose and sample were poured into 10 ml *E*. *coli* prepared media and incubated for 24 to 48 hrs at 35.5^°^C. Subsequently, for completed phase, tubes positive for *E*. *coli* were used to estimate the concentration of *E*. *coli* [39]. The number of *E*. *coli* positive tubes and their estimated concentrations of *E*. *coli* MPN/100 ml are shown in Table 1.

### Ethical considerations

An ethical approval letter was obtained from the Institutional Ethical Review Committee of Wollo University, College of Medicine and Health Sciences. That committee approved both the protocol and the consent forms. Written informed assent and consent were obtained from the caregivers of participating children (both cases and controls), assent on behalf of the participating children, and consent for the caregivers themselves.

The project provided oral rehydration salt treatment for acute diarrheal under-five children. No treatment was given to acute diarrheal under-five children who were already being treated at a health facility during two weeks prior to the survey and those who had already recovered from acute diarrhea during the survey. Caregivers were advised to visit public health facilities when there was no recovery after two days of treatment. This study was conducted according to the protocol and ethical principles of the Declaration of Helsinki by the World Medical Association [43] and also to the principles that govern medical research involving human subjects specified by the Council for International Organizations of Medical Sciences [44].

### Statistical analysis

Data were entered using EpiData Version 3.1 (EpiData Association, Odense, Denmark) software and exported to STATA Version 13.0 statistical software (StataCorp LP, College Station, TX) for data analysis. Before data analysis, data were cleaned using two steps as described elsewhere [45]. Descriptive statistics [n (%)] were carried out for cases and controls, including means (±SD (standard deviations) for continuous variables.

Data analysis was based on our conceptual hierarchical framework shown in Fig 1 using a conditional logistic regression model. The modeling strategy involved estimating unadjusted matched odds ratio (unadjusted mOR) and adjusted mOR of the studied variables with acute diarrhea at 95% CI. Thus, data analysis of unadjusted mOR and adjusted mOR was employed only for the fully paired data by conditional logistic regression model, while taking into account the matched analysis of cases and controls. Our analysis did not consider children’s ages and neighborhoods since we used both as matching variables for controls with cases during the study design. Variables in Fig 1 were classified as distal (block 1), intermediate (blocks 2, 3, 4), and proximate (block 5) determinants according to our hierarchical framework, which was developed based on our study context and similar methods described elsewhere [46, 47].

Bivariate analysis identified variables associated with acute diarrhea at p<0.05. From the bivariate analysis, variables that had a significance level of p<0.2 in each block were retained for inclusion into the multivariable analysis of hierarchical conditional logistic regression consisting of five models (models 1, 2, 3, 4 and 5) and a final model based on the five blocks of variables in Fig 1. The purpose of each model was to identify potential confounders in a step-by-step fashion, by taking into account the levels of influence as distal, intermediate, and proximate factors. The variables that remained significantly associated with acute diarrhea at p<0.2 in the multivariable analysis of each model were included in the subsequent model [48, 49]. The final model includes only variables with a p<0.2 from model 5. In the final model, variables at p<0.05 were considered as statistically significant and independently associated with acute diarrhea.

## Results

### Socio-demographic characteristics of cases and controls

The response rate for fully matched paired data from the survey and water sample was 190 matched pairs (95.5%) and 48 matched pairs (96.0%), respectively. Nine cases did not match any controls. No water samples were taken in one case household that had no stored water for water sampling and from one control household that did not collect water from a piped water supply (Fig 2).

The mean age of the caregivers was 28.4 (±5.5) years for cases and 29.8 (±6.2) for controls. The mean monthly household income was $36.1 (±22.8) US for case households and $52.2 (±27.4) US for control households (Table 2). Out of 190 cases, 54.2% were males and 45.8% were females, whereas out of 570 controls, 52.2% were males and 47.8% were females (Table 3). The age distribution of cases and controls was 136 (17.9%) between 0–11 months old, 208 (27.4%) between 12–23 months old, and 416 (54.7%) between 24–59 months old.

**Table 2:** Characteristics [mean (±SD)] of case and control households in Addis Ababa slums, Ethiopia, November to December, 2014.

	Case households (N = 190)		Control households (N = 570)	
Variables	Mean	SD	Mean	SD
Age of caregivers (years)	28.4	5.5	29.8	6.2
Monthly household income ($US^1^)	36.1	22.8	52.2	27.4
Household size (persons)	5.4	1.9	5.4	1.9
Intermittent piped water supply (days water not available per two weeks)	4.3	1.7	3.9	2.7
Daily per capita water consumption (l/c/d)	11.5	4.9	14.6	5.1
Water storage duration (days)	3.1	2.2	2.2	2.0

SD, Standard deviations; l/c/d, liter per capita per day; $US, United States Dollars.

^1^The average exchange rate of $1 US = 20.0 ETB (Ethiopia birr) from November to December, 2014.

**Table 3:** Bivariate analysis of socio-demographic characteristics with acute diarrhea among under-five children in Addis Ababa slums, Ethiopia, November to December, 2014.

	Cases (N = 190)	Controls (N = 570)	
Variables in block 1	n (%)	n (%)	Unadjusted mOR (95% CI)^§^
Age of caregivers (years)			
<25	39(20.5)	98(17.2)	1.5(0.9–2.7)
25–34	119(62.6)	350(61.4)	1.3(0.8–2.1)
>34	32(16.9)	122(21.4)	1
Monthly household income ($US)			
Less than $50 US	133(70.0)	268(47.0)	3.0(2.1–4.4)
$50 US or above	57(30.0)	302(53.0)	1
Housing ownership			
Rent or other*	175(92.0)	515(90.4)	1.3(0.7–2.3)
Own house	15(8.0)	55(9.6)	1
Education of caregivers			
Did not attend formal or informal education**	83(43.7)	145(25.4)	2.4(1.7–3.4)
Attended formal and/or informal education	107(56.3)	425(74.6)	1
Household size			
6 or more persons	84(44.2)	237(41.6)	1.1(0.8–1.6)
2–5 persons	106(55.8)	333(58.4)	1
Marital status of caregivers			
Unmarried	62(32.6)	101(17.7)	2.3(1.6–3.4)
Married	128(67.4)	469(82.3)	1
Employment of caregivers			
No	89(46.8)	258(45.3)	1.1(0.8–1.5)
Yes	101(53.2)	312(54.7)	1
Child gender			
Male	103(54.2)	298(52.3)	1.1(0.8–1.5)
Female	87(45.8)	272(47.7)	1
Number of under-five children per household			
2–4 children	44(23.2)	119(20.9)	1.2(0.8–1.7)
1 child	146(76.8)	451(79.1)	1

Unadjusted mOR, Unadjusted matched odds ratio; CI, Confidence interval.

*Houses that were illegally constructed and had no owner or houses temporarily provided by families to relatives or other persons.

**Unable to read or write based on self-reporting.

^§^Denotes unadjusted mOR using 95% confidence interval from bivariate conditional logistic regression analysis in matched case-control pairs.

1 Reference category.

### Intermittent piped water supplies and daily per capita water consumption

Over two-thirds (69.7%) of control households and 63.7% of case households obtained water from private taps, whereas 30.3% of control households and 36.3% of case households fetched water from public taps (Table 4). Overall, mean duration of intermittent piped water supply during the two weeks before the survey was 4.3 days in case households, and 3.9 days in control households. Average daily per capita water consumption per person was 11.5 and 14.6 liters among case and control households, respectively (Table 2).

**Table 4:** Bivariate analysis of piped water supplies and water-handling practices with acute diarrhea among under-five children in Addis Ababa slums, Ethiopia, November to December, 2014.

	Cases (N = 190)	Controls (N = 570)	
Variables in blocks 2, 3, 4, 5	n (%)	n (%)	Unadjusted mOR (95% CI)^§^
Piped water supply location			
Outside house compound	131(68.9)	296(51.9)	2.6(1.7–3.9)
Inside house compound and/or home	59(31.1)	274(49.1)	1
Piped water supply type			
Public tap	69(36.3)	173(30.3)	1.6(0.9–2.4)
Private tap	121(63.7)	397(69.7)	1
Intermittent piped supply of water			
Yes	167(87.9)	291(51.0)	11.1(6.4–19.4)
No	23(12.1)	279(49.0)	1
Purchasing water from piped supply			
Yes	138(72.6)	310(54.4)	2.9(1.9–4.5)
No*	52(37.4)	260(45.6)	1
Mouth size of water storage container			
Wide mouthed	22(11.6)	30(5.3)	6.3(3.0–13.4)
Wide and narrow mouthed	30(15.8)	192(33.7)	3.4(2.0–5.6)
Narrow mouthed	138(72.6)	348(61.0)	1
Type of vessels used for retrieving water from water storage container			
Without handle	118(62.1)	87(15.3)	15.5(9.3–25.9)
With and without handle	28(14.7)	75(13.1)	4.7(2.6–8.7)
With handle	44(23.2)	408(71.6)	1
Water storage duration during two weeks before the survey			
Above 3.5 days	71(37.4)	136(23.9)	3.8(2.2–6.6)
3.5 days and below	119(62.6)	434(76.1)	1
Cleaning of water storage container during two weeks before the survey			
Not cleaned	17(8.9)	18(3.2)	3.3(1.6–6.7)
Cleaned at least once	173(91.1)	552(96.8)	1
Daily per capita water consumption (l/c/d)			
Less than 20 liters	165(86.8)	409(71.8)	3.1(1.9–5.2)
20 liters or more	25(13.2)	161(28.2)	1

Unadjusted mOR, Unadjusted matched odds ratio; CI, Confidence interval; *E*. *coli*, *Escherichia coli*.

1 Reference category.

^§^Denotes unadjusted mOR using 95% confidence interval in bivariate conditional logistic regression analysis from matched case-control pairs.

^*^Households that did not buy water during every water collection time; they paid for water consumption with the house rent or by the volume of water consumed every month as billed by the water authority.

### Household water-handling practices

Households stored water for a minimum of half a day and a maximum of 9.5 days during the two weeks before the survey. The average water storage duration was 3.1 days for case households and 2.2 days for control households during the two weeks before the survey (Table 2). Water was stored for 3.5 days or less during that time period by 62.6% of the case households and 76.1% of the control households. Characteristics of household water-handling practices and the results of the bivariate analysis of acute diarrhea are summarized in Table 4.

### *E*. *coli* contamination at piped water supplies and in household stored water

In the 192 households where water samples were taken from their piped water, the bacteriological quality was <2.2 *E*. *coli* MPN/100 ml in 66.7% of the case households and 62.5% of the control households; and 2.2 *E*. *coli* MPN/100 ml in 29.2% of the case households and 31.2% of the control households. Overall, further contamination by *E*. *coli* at POU in household stored water was found in most 40 (83.3%) of the case households and 75 (52.1%) of the control households. In the bivariate analysis, contamination of piped water supplies by *E*. *coli* was not associated with acute diarrhea (unadjusted mOR = 0.7; 95% CI: 0.3–1.5) (Table 4). The stored water of 42 (87.5%) case households and 112 (77.8%) control households did not meet the *E*. *coli* standards (no detectable *E*. *coli* positive tubes out of the five tubes [<2.2 *E*. *coli* MPN/100 ml]) (Table 5).

**Table 5:** Comparison of number of *E*. *coli* MPN/100 ml between piped water supply and household stored water among case and control households in Addis Ababa slums, Ethiopia, November to December, 2014.

	Cases (N = 48)	Controls (N = 144)	Compliance with drinking water standards	Unadjusted mOR (95% CI)^§^
Water quality variable (blocks 3, 5)	n (%)	n (%)		
*E*. *coli* in piped water supply^+^				0.7(0.3–1.5)
<2.2 MPN/100 ml ^ǂ^	32(66.7)	90(62.5)	Yes	1
2.2 MPN/100 ml	14(29.2)	45(31.2)	No	0.9(0.4–1.8
5.1 MPN/100 ml	2(4.1)	9(6.3)	No	0.6(0.1–3.0)
*E*. *coli* in household stored water^+^				
<2.2 MPN/100 ml ^ǂ^	6(12.5)	32(22.2)	Yes	1
2.2 MPN/100 ml	2(4.2)	59(41.1)	No	0.1(0.0–1.1)
5.1 MPN/100 ml	4(8.3)	19(13.2)	No	1.3(0.3–5.7)
9.2 MPN/100 ml	9(18.7)	13(9.0)	No	3.7(0.9–15.2)
16.0 MPN/100 ml	15(31.3)	11(7.6)	No	9.0(2.3–35.6)
>16.0 MPN/100 ml	12(25.0)	10(6.9)	No	6.3(1.7–23.4)
POU *E*. *coli* contamination of household stored water^+^				
Yes	40(83.3)	75(52.1)	No	5.6(2.2–13.9)
No	8(16.7)	69(47.9)		1

mOR, matched odds ratio; CI, confidence interval; *E*. *coli*, *Escherichia coli;* MPN, Most Probable Number.

^§^Denotes unadjusted mOR using 95% confidence interval from bivariate conditional logistic regression analysis from matched case-control pairs.

^ǂ^Indicates no *E*. *coli* positive tubes detected out of the five tubes which met the standard of drinking water quality (<2.2 *E*.*coli* MPN/100 ml) [41, 42].

^+^Denotes the denominator differs due to data were only from 25% of matched pair sample size.

1 Reference category.

### Multivariable hierarchical conditional logistic regression analysis

In the multivariable analysis, we found that intermittent piped water supplies, POU contamination of household stored water by *E*. *coli*, water retrieval from household water storage containers by using handle-less vessels, and water retrieval from household water storage containers by interchangeably using vessels both with and without a handle were independently associated with acute diarrhea among under-five children in Addis Ababa slums.

Our main findings show that the odds of developing acute diarrhea among under-five children in households with intermittent water supplies were 4.8 times (adjusted mOR = 4.8; 95% CI: 1.3–17.8) higher than in households that had continuous water supply. The likelihood of developing acute diarrhea among under-five children in households that had POU contamination of stored water by *E*. *coli* was 3.3 times (adjusted mOR = 3.3; 95% CI: 1.1–10.1) higher than in households that had no POU contamination of household stored water. Water retrieval from water storage containers using vessels without a handle (adjusted mOR = 16.3; 95% CI: 4.4–60.1) and with and without a handle (adjusted mOR = 5.4; 95% CI: 1.1–29.1) was also significantly associated with acute diarrhea (Table 6).

**Table 6:** Factors independently associated with acute diarrhea among under-five children in Addis Ababa slums in the multivariable analysis.

Variables	Model 1	Model 2	Model 3	Model 4	Model 5	Final model Adjusted mOR (95% CI)^1^
Age of caregivers (years)						
<25	1.4(0.8–2.6)					
25–34	1.3(0.8–2.2)					
>34	1					
Monthly household income						
Less than $50 US	2.4(1.6–3.6)*	2.3(1.5–3.5)*	2.4(1.5–3.8)*	1.9(1.1–3.3)*	1.2(0.3–5.0)	
$50 US or above	1	1	1	1	1	
Education of caregivers						
Did not attend formal or informal education	2.0(1.4–3.0)*	1.9(1.3–2.9)*	1.8(1.2–2.9)*	2.0(1.2–3.3)*	1.1(0.4–3.0)	
Attended formal and/or informal education	1	1	1	1	1	
Marital status of caregivers						
Unmarried	1.9(1.3–2.8)*	1.8(1.2–2.8)*	2.1(1.3–3.4)*	2.6(1.5–4.5)*	0.9(0.2–3.9)	
Married	1	1	1	1	1	
Piped water supply location						
Outside house compound		2.6(1.5–4.3)*	1.8(0.6–5.4)			
Inside house compound and/or home		1	1			
Piped water supply type						
Public water		1.5(0.8–2.6)**	1.3(0.7–2.5)			
Private tap water		1	1			
Intermittent piped water supply						
Yes			12.2(6.6–22.4)*	7.2(3.5–14.7)*	4.9(1.2–20.2)*	4.8(1.3–17.8)*
No			1	1	1	1
Purchasing water from piped water supply						
Yes			1.7(0.5–5.2)			
No			1			
Mouth size of water storage container						
Wide mouthed				5.9(2.1–16.5)*	4.5(0.5–39.4)**	3.6(0.5–26.4)
Wide and narrow mouthed				2.4(1.3–4.6)*	1.5(0.4–5.7)	1.4(0.4–4.9)
Narrow mouthed				1	1	1
Type of vessels used for retrieving water from water storage container						
Without handle				9.7(5.3–17.7)*	15.0(3.7–60.7)*	16.3(4.4–60.1)*
With and without handle				3.2(1.5–6.6)*	4.9(0.8–29.5)**	5.4(1.1–29.1)*
With handle				1	1	1
Water storage duration during two weeks before the survey						
>3.5 days				2.2(0.9–5.1)**	0.7(0.1–3.2)	
≤3.5 days				1	1	
Cleaning of water storage container during two weeks before the survey						
Not cleaned				0.7(0.2–1.8)		
Cleaned at least once				1		
POU contamination of household stored water by *E*. *coli*^+^						
Yes					3.8(1.1–12.8)*	3.3(1.1–10.1)*
No					1	1
Daily per capita water consumption (l/c/d)						
Less than 20 liters					1.6(0.6–4.4)	
20 liters or more					1	

Adjusted mOR, adjusted matched odds ratio; CI, confidence interval; *E*. *coli*, *Escherichia coli;* POU, Point-of-Use

Model 1, Includes variables that had p<0.2 from the bivariate analysis of block 1 variables.

Model 2, Includes variables that had p<0.2 from model 1 and variables that had p<0.2 from bivariate analysis of block 2 variables.

Model 3, Includes variables that had p<0.2 from model 2 and variables that had p<0.2 from bivariate analysis of block 3 variables.

Model 4, Includes variables that had p<0.2 from model 3 and variables that had p<0.2 from bivariate analysis of block 4 variables.

Model 5, Includes variables that had p<0.2 from model 4 and variables that had p<0.2 from bivariate analysis of block 5 variables.

Final model; Includes variables that had p<0.2 from model 5 (Intermittent piped water supply, mouth size of water storage container, type of vessels used for retrieving water from water storage container and POU contamination of household stored water by *E*. *coli*).

^1^Denotes adjusted mOR from the final model using 95% confidence interval from multivariable hierarchical conditional logistic regression analysis from matched case-control pairs.

*Statistically significant at p<0.05.

**Statistically significant at p<0.2.

^+^Denominator differs because data were from only 25% of the matched pair sample.

1 Reference category.

## Discussion

We used a community-based matched case-control study design supported by *E*. *coli* laboratory analysis of water from piped water supply and household stored water to examine the relationship betewen intermittent piped water supplies, POU contamination of household stored water by *E*. *coli* with acute diarrhea among under-five children in slums of Addis Ababa. We found that intermittent supply from piped water, POU contamination of household stored water by *E*. *coli*, use of vessels without handles and interchangeable use of vessels both with and without handles for retrieving water from household water storage containers were independently associated with acute diarrhea.

A matched case-control study in Bamako, Mali, pointed out that continuous availability of drinking water is a protective factor against acute diarrhea among under-five children [22]. Ercumen et al. [10] also found that continuous water supplies were associated with reduced waterborne illness transmissions in the poorer, higher-risk segments of a population. The intermittent piped water supplies found in our study might be a result of a low quantity of water from piped sources and from leaking of the distribution systems. Our finding that deterioration of microbiological quality in household stored water was significantly associated with acute diarrhea is consistent with several other studies [4, 11, 18, 22, 50, 51]. Higher *E*. *coli* contamination in household stored water more than of piped water at the household point of contact found in our study might be due to a lack of washing of water storage containers. The intermittent water supplies might also be a major factor in the practice of home storage of water; and improper water retrieval practices by caregivers may lead to *E*. *coli* contamination of stored water.

Several other studies revealed that intermittent water supplies lead to water quality deterioration at the household level [12, 13, 22]. A study in a slum in Brazil indicated that contamination of stored water was an important factor in diarrhea transmission [52]. Consistent with our findings, several studies recognized deterioration of the microbiological quality of household stored water [11, 16, 22, 33, 53, 54]. A randomized controlled trial study in rural Bangladesh revealed that safe water storage reduced diarrhea more than did safe storage combined with chlorination [55]. Another study in Dhaka slums in Bangladesh found that microbiological contamination of water was higher at household levels than at primary sources due to poor water storage and unhygienic practices in households [56]. We found that poor retrieval practices were associated with acute diarrhea. The unhygienic water retrieval practices of household water from storage containers may be worsened by use of wide-mouthed water storage containers that allow a hand to enter along with the water-retrieval vessel.

In the multivariable analysis, we found that socio-demographic factors, piped water supply location, cleaning of water storage container, water storage duration, mouth size of water storage containers, daily per capita water consumption, buying water from piped water supplies, and contamination of piped water supplies by *E*. *coli* were not associated with acute diarrhea. This might be due to the similarity of most of the water-handling practices, similar socio-economic status of case and control households, and good quality of piped water delivered by Addis Ababa Water and Sewerage Authority; 94.3% of households had piped water supplies with low risk of *E*. *coli* (2.2 and <2.2 *E*. *coli* MPN/100 ml) contamination. In contrast with our findings, several studies reported that water sources located away from homes (at-least 30 minutes walk) [57–59], infrequent cleaning of water storage containers [33], lower daily per capita water consumption [60], and low education status of caregivers [61] were significantly associated with acute diarrhea.

Our study draws its strength from using a mixed approach of a community-based survey and *E*. *coli* analysis of water supply from piped water supplies and stored household water. In addition, we used a matched case-control study design where the matching helped to control for potential confounders, further strengthening our conclusion and recommendations. This study was conducted during a period with almost very little rainfall, which may have resulted in better bacteriological water quality from piped water supplies than during the rainy season, when fecal contamination tends to degrade surface and ground water microbial quality [62]. On the other hand, the frequency of stoppages of the intermittent water supply might decrease during the rainy seasons because of the greater availability of surface water for the water treatment plant. Therefore, further studies that consider intermittent water supply, bacteriological quality of piped water supplies, and water borne diseases among under-five children during the rainy seasons in Addis Ababa slums are encouraged.

Unfortunately, since our laboratory analysis of *E*. *coli* using a five tubes MPN/100 ml method could only roughly indicate the most probable number of *E*. *coli* in the water, comparing the quality of POU household stored water and piped water supplies based on WHO contamination risk levels of *E*. *coli* [no risk (<1 colony-forming units (CFU)/100 ml), low risk (1–10 CFU/100 ml), moderate risk (11–100 CFU/100 ml), and high risk and above (>100 CFU/100 ml)] [41] is difficult. Therefore, we encourage further studies using other methods that are able to more accurately quantify the concentration of *E*. *coli*. Limitations of the study also include our failure to study the mediated effect of variables using mediation models from the hierarchical conceptual framework we developed because we did not analyze *E*. *coli* occurrence in all case and control household piped water supplies and stored household water. However, we consider the findings to be valid since we analyzed *E*. *coli* distributions based on the recommended sample size and systematically selected every fourth case household and its matched controls. Future research should elaborate the pathways (direct and indirect effect) for fecal contamination of household stored water received from improved piped water supplies. In order to ensure a low confidence interval of the variables in the adjusted analysis of the final model, these studies should use a comparable sample size for the household survey of water samples for *E*. *coli* laboratory analysis. In addition, our retrospective study could not determine causality and reverse causation, pointing to the need to carry out prospective cohort studies to examine causal relationships.

## Conclusion

Although provision of potable piped water by municipalities is frequently reported to reduce acute diarrhea in under-five children in slums, our data show that this is not a sufficient condition to prevent acute diarrhea. This is largely due to intermittent water supplies and POU contamination arising from poor hygienic practices such as using vessels that have no handle when retrieving water from storage containers. We conclude that continuous availability of piped water supplies and education about proper water retrieval methods from household water storage containers can effectively reduce POU contamination of water at the household level. A water supply policy that focuses continuous availability of piped water supplies and proper water handling practices at the household level may reduce the practice of household water storage and prevent microbiological contamination of stored water, and in turn reduce diarrhea risk among under-five children in slums of Addis Ababa and other urban slums in Ethiopia. In addition, promotion of household water treatment practices is also highly encouraged as an alternative solution until the Addis Ababa Water and Sewerage Authority is able to deliver continuously available piped water supplies.

## Supporting information

S1 File Procedures for water sampling methods at household stored water and piped water supplies and water sample transportation to laboratory rooms. (DOCX)

## Abbreviations

Adjusted mOR: Adjusted matched odds ratio
Unadjusted mOR: Unadjusted matched odds ratio
CI: Confidence interval
*E. coli*: *Escherichia coli*
l/c/d: liter per capita per day
MPN: Most Probable Number
POU: Point-of-Use
$US: United States Dollars
WHO: World Health Organization
