# Supplementary material for: Piped water supply interruptions and acute diarrhea among under-five children in Addis Ababa slums, Ethiopia: A matched case-control study
Source: PLoS One. 2017 Jul 19;12(7):e0181516. doi: 10.1371/journal.pone.0181516 (PMC5517045; doi:10.1371/journal.pone.0181516)
Supplement: S1 File — (DOCX) [file pone.0181516.s001.docx]

**Water sampling methods and transporting water samples to laboratory rooms**

Water samples were taken according to WHO water sampling procedures ([WHO, 1997](#_ENREF_253)), as follows:

**1) Drinking water sample from taps, public water (*Bono* and tanker) for *E. coli* analysis**

- Use the 250 ml sterile capacity polyethylene bottles supplied by the laboratory for bacteriological analyses. If the sterile seal is broken, do not use
- Wash your hands carefully with soap and water before collecting the sample
- Remove the aeration screen from the tap
- and water before collecting the sample
- Remove the aeration screen from tap
- Disinfect the end of the tap faucet with a bleach solution or other disinfecting agent
- Allow the water to run for two minutes
- Take the cap off the bottle and hold the cap in one hand and the bottle in the other. **Never rinse the bottle.** The bottle contains a thio-sulfate tablet to neutralize any chlorine and prevent ongoing bacteria growth
- Collect the sample from the drinking water outlet of the tap and public tap water through pouring
- Carefully fill the bottle within 6-7 mm [¼ inch] of the top through pouring
- Replace the cap to the bottle without touching the inside of the cap or the mouth of the bottle

**2)  Drinking water sample from water storage container for *E. coli* analysis**

- Use the 250 ml capacity sterile polyethylene bottles supplied by the lab for bacteriological analyses. If the sterile seal is broken, do not use.
- Wash your hands carefully with soap and water before collecting the sample
- Disinfect the end and side of the covered water storage container outlet with a bleach solution or other disinfecting agent
- Remove the covered materials
- Allow the stored water to run for two minutes
- Take the cap off the bottle and hold the cap in one hand and the bottle in the other. **Never rinse the bottle.** The bottle contains a thio-sulfate tablet to neutralize any chlorine and prevent ongoing bacteria growth
- Collect the sample from the drinking water storage container outlet through pouring
- Carefully fill the bottle within 6-7 mm [¼ inch] of the top through pouring
- Replace the cap to the bottle without touching the inside of the cap or the mouth of the bottle

**3] Drinking water sample shipping instructions for *E. coli* analysis during transportation to laboratory room**

- Clearly print sample code and sample location on the sample bottle.
- Place the sample bottle with the correct requisition. Secure the lid with tap.
- Place the sample with ice-box [insulated cooler].
- Place enough approved ice packs in the cooler so the sample will stay cool but will not freeze during transportation to the lab.
- Bring the sample to the laboratory within two hours for laboratory analysis of two to four hour.
